# Effectiveness of Telemedicine for Reducing Cardiovascular Risk: A Systematic Review and Meta-Analysis

**DOI:** 10.3390/jcm12030841

**Published:** 2023-01-20

**Authors:** Jesús Jaén-Extremera, Diego Fernando Afanador-Restrepo, Yulieth Rivas-Campo, Alejandro Gómez-Rodas, Agustín Aibar-Almazán, Fidel Hita-Contreras, María del Carmen Carcelén-Fraile, Yolanda Castellote-Caballero, Raúl Ortiz-Quesada

**Affiliations:** 1 Department of Health Sciences, Faculty of Health Sciences, University of Jaén, 23071 Jaén, Spain; 2 Faculty of Health Sciences and Sport, University Foundation of the Área Andina, Pereira 660004, Colombia; 3 Faculty of Human and Social Sciences, University of San Buenaventura, Santiago de Cali 760016, Colombia; 4 Department of Anatomy and Embryology, Faculty of Medicine, University of Granada, 18071 Granada, Spain; 5 Instituto de Investigación Biosanitaria de Granada (ibs.GRANADA), 18014 Granada, Spain

**Keywords:** telemedicine, risk factors for heart disease, hypertension, overweight, diabetes mellitus, sedentarism

## Abstract

Background: Cardiovascular diseases are the leading cause of death globally. There are six cardiovascular risk factors: diabetes, hypertension, hypercholesterolemia, overweight, sedentary lifestyle and smoking. Due to the low attendance of healthy people in the health system, the use of telemedicine can influence the acquisition of a heart-healthy lifestyle. Objective: this systematic review and meta-analysis aimed to determine the effectiveness of telemedicine and e-health in reducing cardiovascular risk. Methods: A systematic review and meta-analysis were carried out using the PubMed, Scopus, Cinhal and WOS databases. Randomized controlled studies between 2017 and 2022 in which telemedicine was used to reduce any of the risk factors were included. The methodological quality was assessed using the “PEDro” scale. Results: In total, 763 studies were obtained; after the review, 28 target articles were selected and finally grouped as follows: 13 studies on diabetes, six on hypertension, seven on obesity and two on physical activity. For all of the risk factors, a small effect of the intervention was seen. Conclusions: although the current evidence is heterogeneous regarding the statistically significant effects of telemedicine on various cardiovascular risk factors, its clinical relevance is undeniable; therefore, its use is recommended as long as the necessary infrastructure exists.

## 1. Introduction

Cardiovascular diseases (CVDs) remain the most common cause of death in Europe. In people aged below 70 years, deaths from CVD are considered premature. It is estimated that CVD deaths in Europe alone exceed 60 million [1]. The analysis of the causes of mortality makes it possible to better direct resources and efforts from public health policies [2,3]. In developed countries, these policies focus on strengthening primary care, identifying people at higher risk and implementing strategies before the event occurs [4].

Unlike risk markers (sex, age, genetic predisposition, etc.), which are inherent to the person and, therefore, not modifiable, risk factors (RFs) are acquired conditioning factors that predispose a person or population to suffer an event but that can be intervened [3].

Based on epidemiological studies carried out in the 1950s, it is well known that part of the morbimortality of cardiovascular diseases is avoidable, since it is associated with cardiovascular risk factors (CVRFs). With the Framingham study [5], six main CVRFs were identified, recognizing that people who smoke (daily consumption of at least 20 cig/day), are obese (BMI ≥ 30), are sedentary (no physical activity in their free time, occupying this time with activities that do not include movement), are diabetic (in DM, Hbg > 6.5 and/or antidiabetic treatment), with hypertension (BP ≥ 140/90 mmHg and/or antihypertensive treatment) or with hypercholesterolemia (values greater than 221 mg/dL in total cholesterol, 141 mg/dL in LDL and 135 mg/dL in triglycerides) are more likely to suffer or die from cardiovascular disease [5,6].

Given that not all CVRFs have the same importance in causing CVD, different mathematical models have been designed to calculate the cardiovascular risk (CVR) as accurately as possible. The most reasonable and cost-effective method is based on prioritization by estimating the cardiovascular risk of asymptomatic patients. Among these are the REGICOR tables, constructed from the Framingham equation and validated for the entire Spanish population for use in primary care, estimating the coronary risk according to the presence of CVRFs. In these tables, patients with a risk greater than 20% are considered to be at high risk [7].

Currently, it is difficult for health care providers to reach healthy populations at risk due to the presence of economic, geographic and/or access limitations; therefore, the transmission of health information through information and telecommunication technologies that allow the administration of clinical, administrative and educational services at the patient’s home (telemedicine) [8], either through teleconsultation [9], tele-education [10], telemonitoring [11] or tele-surgery [12], is becoming increasingly popular.

Clinical practice guidelines indicate multidisciplinary interventions, acting on CVRFs in a comprehensive manner, to promote a cardio-healthy lifestyle [3]. These interventions can be adapted to new technologies, bringing health care closer to the patient, trying to reduce CVR through health education programs focused on addressing CVRFs as well as using pharmacological measures to control hypertension, hypercholesterolemia, diabetes, etc. [13].

Governments in developed countries are adopting an increasing number of strategies that focus on telemedicine or e-health for people with chronic diseases, which make up the bulk of cardiovascular risk. However, it is still unclear whether these interventions are effective at mitigating the risk associated with chronic diseases [8]. Therefore, the aim of this systematic review and meta-analysis were to determine the effectiveness of telemedicine-based interventions at reducing cardiovascular RFs.

## 2. Materials and Methods

This systematic review and meta-analysis aimed to evaluate the effectiveness of telemedicine and e-health at reducing cardiovascular risk, segregating this reduction in the following modifiable RFs: diabetes, hypertension, hypercholesterolemia, overweight/obesity and physical activity [3]. The review and meta-analysis were conducted under the guidelines of the PRISMA 2020 document [14] and the prespecified protocol is registered in PROSPERO (CRD42022365395). In addition, the methodological recommendations of the “Cochrane Manual for the Elaboration of Systematic Reviews of Interventions” proposed by Higgins et al. [15] were considered.

### 2.1. Sources of Information

A literature search was conducted between September and October 2022 in the PubMed, Scopus, Cinhal and Web of Science databases.

### 2.2. Search Strategy

Different keywords were used connected in the following search equation: ((Telemedicine OR Remote Consultation) AND (Heart Disease Risk Factors OR hypertension OR obesity OR overweight OR cholesterol OR Hypercholesterolemia OR diabetes mellitus OR smoking OR exercise OR sedentary behavior) AND (middle aged)).

### 2.3. Selection Criteria

The articles had to meet the following inclusion criteria: (1) studies referring to telemedicine and e-health; (2) randomized clinical trials; (3) published within the last 5 years (2017–2022); (4) studies that focused on the general population, excluding ethnic minorities and populations with health problems not relevant to those assessed as CVRFs (i.e., diabetes, hypertension, obesity/overweight, hypercholesterolemia, tobacco use, exercise and sedentary lifestyle).

### 2.4. Study Selection Process

The selection of articles was carried out using the Rayyan virtual tool (https://rayyan.qcri.org/welcome, accessed on 5 September 2022) [16] which made it possible to identify and eliminate duplicate articles. The title and abstract were read, and the articles that met the inclusion criteria were filtered. Two of the authors determined their choice without knowing the verdict of the other researcher, and in the case of divergent decisions, a third author defined the relevance of its inclusion of the study.

### 2.5. Data Extraction

The main variables of this review and meta-analysis focused on the measurement of CVRFs with glycosylated hemoglobin, blood pressure, weight and measurement of vigorous/moderate physical activity in minutes per week. We included data on the authors, year of publication, country, population characteristics, characteristics of the intervention and the control group, type of telemedicine and follow-up time (i.e., 3 months, 6 months or 1 year) as well as the results obtained.

### 2.6. Methodological Quality Assessment

For the assessment of the quality of the articles chosen for the review, the PEDro scale was used, which is one of the scales with the highest reliability index for the evaluation of the methodological quality of different publications. This scale is composed of eleven items, which evaluate the internal and external validity and statistical support of the articles [17,18]. The first item is the only one that comprises external validity and is not included in the final sum. Each of the remaining items are scored from zero to one, depending on whether it appears (one) or not (zero) in the publication. A score between 0 and 3 was considered “Poor” quality; between 4 and 5 was “Fair”, 6–8 was “Good” and >9 was “Excellent” [19].

### 2.7. Analytic Decisions for Meta-Analysis

A meta-analysis of the data was performed for the mean and standard deviation of the changes over time of the observed variables by applying a random effects model. When SD was not available, the 95% CI was used.

The results of the meta-analysis are shown as a forest plot, showing the first author, the date of publication, the sample size, the individual effects with (Hedge’s g), and the overall effect with the 95% CI, as well as the *p*-value associated with the statistic. To address possible publication bias, graphical analyses were performed using funnel plots and their distribution.

## 3. Results

### 3.1. Selection of the Studies

**Figure 1:**
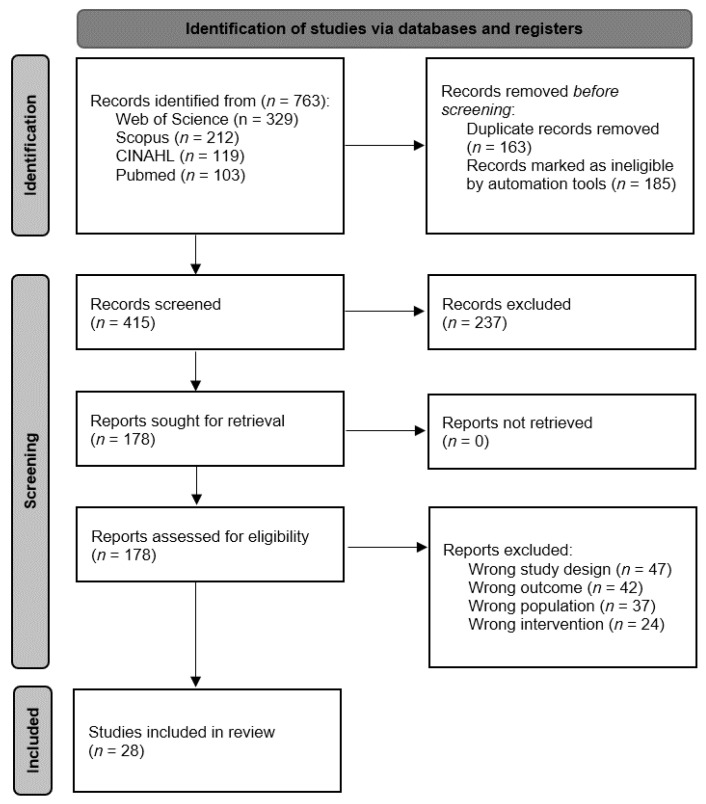
Flow diagram of the study selection process.

A complete search was carried out in different databases, resulting in a total of 763 articles. Subsequently, duplicate articles were removed, and a filter was applied through automation tools leaving a total of 415 articles that were screened. Once the screening was completed, 178 articles were assessed for eligibility, where only 28 articles [20,21,22,23,24,25,26,27,28,29,30,31,32,33,34,35,36,37,38,39,40,41,42,43,44,45,46,47] met the inclusion criteria (Figure 1).

### 3.2. Methodological Quality

The methodological quality was assessed through PEDro. Six of the studies [25,36,41,42,43,46] were obtained on the PEDro website, while the other twenty-two [20,21,22,23,24,26,27,28,29,30,31,32,33,34,35,37,38,39,40,44,45,47] were calculated manually. Of all the included articles, only five articles presented a methodological quality different from Good; one was Poor [41] and another was Fair [45], while three had an Excellent methodological quality [20,24,37] (Table 1).

### 3.3. Characteristics of the Studies

The articles included in this systematic review and meta-analysis were all randomized controlled clinical trials published in the United States [20,21,28,38,40,41,44,45], Australia [29,42,46,47], United Kingdom [23,32], Canada [34,37], Spain [39,43], Malaysia [24], Belgium [22], Iran [30], South Korea [26], Germany [27], Turkey [25], France [31], China [33] and Taiwan [35]. 

A total of 5460 people, aged between 18 and 75 years, participated in the included studies. All participants had to have one of the following conditions: diabetes [20,21,22,23,24,25,26,27,28,29,30,31,32], hypertension [33,34,35,36,37,38], overweight [39,40,41,42,43,44,45] or being sedentary [46,47]. The duration of the interventions ranged from one month to two years. 

### 3.4. Study Results

#### 3.4.1. Diabetes

Among the thirteen articles [20,21,22,23,24,25,26,27,28,29,30,31,32] that evaluated the effects of the use of new technologies on diabetes (Table 2), only four obtained statistically significant results at some point during follow-up [22,27,31,32]. Buysse et al. [22] were able to contrast that after 2 years of intervention, HbA1c decreased −0.40% (SD = 1, *p* = 0.002). These findings are congruent with those of von Storch et al. [27], who obtained a decrease of −0.46% (SD = 0.75, *p* < 0.001) with a modest effect size (Cohen’s d = 0.51) at 3 months after treatment; however, when they compared the pre-intervention data with the data at 6 months, they observed that the difference was reduced to −0.36% (SD = 0.17, *p* < 0.03) with a small effect size (Cohen’s d = 0.40). Franc et al. [31] evidenced that following a telemedicine-based intervention, HbA1c decreased −0.51% (95% CI: 0.30–0.73, *p* < 0.001). Finally, Parsons et al. [32] observed a −0.47% (95% CI: 0.25–0.68, *p* = 0.001) reduction in HbA1c at three months; at six months the reduction was −0.71% (95% CI: 0.44–0.98, *p* = 0.001); and at one year, it reached −0.87% (95% CI: 0.55–1.19, *p* = 0.001).

**Figure 2:**
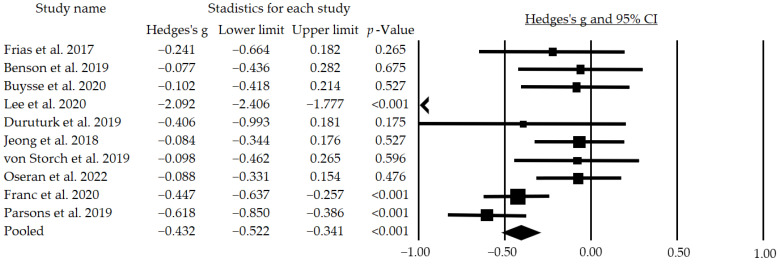
Effects of telemedicine on HbA1c in people with diabetes. The black box represents the point estimate for the respective study, while the size of the box represents the population size and the horizontal line is the 95% CI. The diamond-shaped figure represents the estimated point of the mean effect size. The arrow represents that the point estimate for this study is less than −1.00 [19,20,21,23,24,25,26,27,30,31].

In addition, ten of the articles could be included in the meta-analysis, showing a significant but small mean effect size of g = −0.432 (95% CI: −0.522–−0.341; *p* < 0.001) (Figure 2). In addition, following a subgroup analysis excluding the studies that did not blind participants, we found a moderate and significant mean effect size of g = −0.538 (95% CI: −0.987–−0.089; *p* = 0.019).

#### 3.4.2. Hypertension

Six of the articles [33,34,35,36,37,38] measured the effects of telemedicine, telemonitoring, telecare or the implementation of APPs on systolic and diastolic pressure (Table 3), finding statistically significant changes in four of them [33,35,36,38]. Gong et al. [33] observed a statistically significant (*p* < 0.05) improvement in systolic (−8.90 mmHg, SD = 6.40) and diastolic (−7.00 mmHg, SD = 6.10) blood pressure following intervention. However, Kao et al. [35] evidenced a statistically significant reduction only in systolic blood pressure at three months (−5.28 mmHg, x^2^: 15.2) and at six months (−7.99 mmHg, x^2^: 30.9), while changes in diastolic blood pressure were not statistically significant at three months (−1.58 mmHg, x^2^: 3.6), and at six months an increase of 0.47 mmHg (x^2^: 0.2) was observed. In the case of Liu et al. [36], a reduction at four months in systolic blood pressure of −10.10 mmHg was observed, with statistical significance. Finally, Meurer et al. [38] contrasted that e-counseling combined with telecare produced a decrease of −9.10 mmHg (SD = 4.10) in systolic pressure.

**Figure 3:**
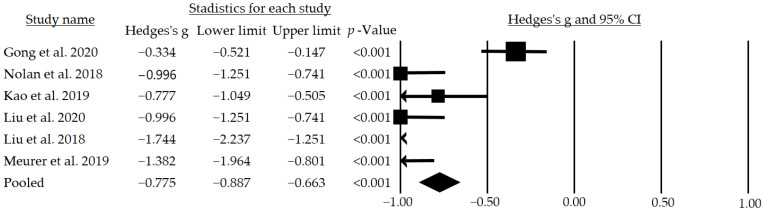
Effects of telemedicine on systolic blood pressure in people with hypertension. The black box represents the point estimate for the respective study, while the size of the box represents the population size and the horizontal line is the 95% CI. The diamond-shaped figure represents the estimated point of the mean effect size. The arrow represents that the point estimate for this study is less than −1.00 [32,33,34,35,36,37].

**Figure 4:**
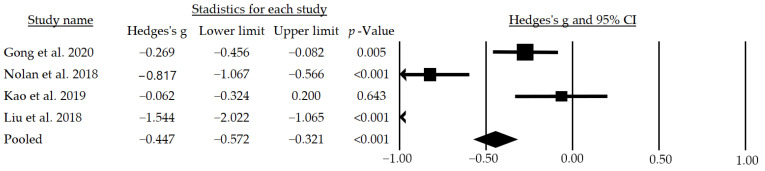
Effects of telemedicine on diastolic blood pressure in people with hypertension. The black box represents the point estimate for the respective study, while the size of the box represents the population size and the horizontal line is the 95% CI. The diamond-shaped figure represents the estimated point of the mean effect size. The arrow represents that the point estimate for this study is less than −1.00 [32,33,34,36].

After the meta-analysis, a moderate and significant mean effect size of g = −0.775 (95% CI: −0.887–−0.663; *p* < 0.001) for systolic blood pressure (Figure 3) and a small but significant mean effect size of g = −0.447 (95% CI: −0.572–−0.321; *p* < 0.001) for diastolic blood pressure were observed (Figure 4). In addition, following a subgroup analysis excluding the studies that did not blind participants, a moderate and significant mean effect size of g = −0.733 (95% CI: −1.252–−0.213; *p* = 0.006) for the systolic pressure was found.

#### 3.4.3. Overweight

Seven studies [39,40,41,42,43,44,45] assessed the effects of telemedicine on body weight in subjects with a BMI > 30 (Table 4); however, only five studies found statistically significant favorable results. In the two studies by Lugones-Sanchez et al. [39,43] and the study by Alencar et al. [41], statistically significant results were observed after 3 months of intervention, achieving a decrease of −2.04 (95% CI = −2.57–−1.50), −1.79 (CI = −2.20–−1.37) and −7.30 kg (SD = −4.40), respectively. Thomas et al. [40] observed that after 12 months of intervention there was a statistically significant small–moderate effect size (r = 0.41) on body weight, while Johnson et al. [45] rejected the null hypothesis, indicating that their intervention had effects on weight reduction, achieving a pre–post difference of −8.23 (SD = 4.50).

**Figure 5:**
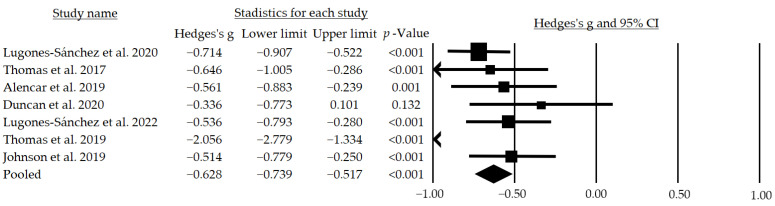
Effects of telemedicine over the body weight in people with overweight. The black box represents the point estimate for the respective study, while the size of the box represents the population size and the horizontal line is the 95% CI. The diamond-shaped figure represents the estimated point of the mean effect size. The arrow represents that the point estimate for this study is less than −1.00 [38,39,40,41,42,43,44].

Finally, through the meta-analysis, a moderate and significant mean effect size of g = −0.628 (95% CI: −0.739–−0.517; *p* < 0.001) was observed (Figure 5). In addition, following a subgroup analysis, when excluding the studies that did not blind participants, a moderate and significant mean effect size of g = −0.728 (95% CI: −1.196–−0.261; *p* = 0.002) was observed, while when excluding those that performed an inadequate follow-up a large and significant mean effect size of g = −0.957 (95% CI: −1.512–−0.401; *p* = 0.001) was obtained.

#### 3.4.4. Sedentarism

Two articles [46,47] evaluated the effects of telemedicine on sedentary lifestyle; however, neither showed statistically significant findings (Table 5). Furthermore, due to the way the variables were presented in both studies, it was impossible to perform a meta-analysis.

### 3.5. Analysis of Publication Bias

The analysis of risk of publication bias with a funnel plot including all 28 articles of the meta-analysis, segregated by risk factor, revealed an expected publication bias due to some studies that altered the effect size because of their low methodological quality. However, when the analysis was performed excluding the articles with low methodological quality, a symmetrical distribution was maintained.

## 4. Discussion

In the clinical practice of health science workers, the needs of patients change day by day. Nowadays, there is a greater need for follow-up and control services for chronic pathologies than for acute care. This chronicity causes an increase in the need for investment that is rarely assimilated by the structure of public health care or public health policies [48]. For this reason, primary health care requires fundamental changes that focus on the control of the RF of pathologies, mainly cardiovascular pathologies due to their high mortality [49].

The most critical RFs for cardiovascular diseases are blood lipid level, high blood pressure, smoking, diabetes, sedentary lifestyle and obesity [50]. Cardiovascular diseases can be prevented by adopting a healthy lifestyle, which should be maintained at all stages of life from childhood to death [51]. A fundamental part of cardiovascular risk management involves the training and coordination of health professionals who are involved in the care and control of the patient, the most representative figure being the primary care nurse. This is because primary care nurses are health promoters and agents of change, with the capacity to promote changes in lifestyles, which is an important strategy for the control of chronic diseases. This strategy, if implemented appropriately, can reduce admissions to emergency departments for acute disorders, improving quality of life and providing patients with more time for useful service to society [3]. Currently, public health policies are aimed at the generation of an integral and multidisciplinary system that allows for the care of patients with chronic pathologies, which would promote equitable medical care, this being one of the pillars of telemedicine [8].

Nowadays, technological advances facilitate the management and control of chronic diseases by means of devices that count steps, measure serum glycemia or even sense heart rate and extrapolate all these data to a computer where a professional can assess and monitor the population, allowing for the promotion of changes or reinforcement of behaviors that have a positive impact on the quality of life of the individual [52]. Many countries have begun to venture into the use of telemedicine and technologies, facilitating access to health systems for a large number of people at a reduced cost. Although multiple authors have now turned their attention to telemedicine [53,54,55], the publications that currently attempt to demonstrate the effectiveness of this intervention present inconsistent results, which can be attributed to the heterogeneity in the design of the intervention itself.

Regarding the methodological quality of the articles analyzed, it was observed that most of them had a good methodological quality. Only two of the articles, focused on overweight, had poor [41] or fair [45] methodological quality; however, this does not seem to be related to the results of the studies, since, although they present statistically significant results, three other studies with good methodological quality also showed statistically significant results. A subgroup analysis showed an increase in the effect size of telemedicine on HbA1c when studies that did not blind their subjects were excluded, from a small mean effect size of g = −0.432 (95% CI: −0.522–−0.341; *p* < 0.001) to a moderate mean effect size of g = −0.538 (95% CI: −0.987–−0.089; *p* = 0.019). In the case of overweight an increase in effect size was observed from a moderate mean effect size of g = −0.628 (95% CI: −0.739–−0.517; *p* < 0.001) to a moderate mean effect size of g = −0.728 (95% CI: −1.196–−0.261; *p* = 0.002) when studies that did not blind participants were excluded, while when studies that did not perform adequate follow-up were excluded, the effect size became a large mean effect size of g = −0.957 (95% CI: −1.512–−0.401; *p* = 0.001).

In patients with diabetes, it has been observed that a decrease of 0.5% or higher in HbA1c is associated with a reduced cardiovascular risk. Although of the studies analyzed only one study [31] achieved a reduction greater than the mentioned value along with a significant difference, in all of them, the HbA1c value was reduced with a small but significant mean effect size (g = −0.432, *p* < 0.001), which allows us to assume that telemedicine is useful for the reduction in cardiovascular risks in patients with diabetes.

Hypertension is currently considered a public health problem worldwide, and it is expected that by 2025, one-third of the entire population will present this pathology [56], making new intervention strategies necessary. Telemedicine, as mentioned above, favors adherence to treatments for chronic diseases; however, the market is saturated with APPs whose efficacy in controlling blood pressure has not been proven through clinical trials [57]. In relation to the findings obtained in this review and meta-analysis, it was observed that most of the articles obtained statistically significant changes in blood pressure with a moderate and significant mean effect size (g = −0.775, *p* < 0.001) for systolic and a small but significant mean effect size (g = −0.447, *p* < 0.001) for diastolic blood pressure; according to the literature, every 2 mmHg lower systolic blood pressure or 1 mmHg lower diastolic blood pressure is associated with a 10% and 7% lower risk of mortality from stroke or ischemic heart disease, respectively [58].

Regarding overweight, the number of cases worldwide is increasing, representing a great risk for public health due to the fact that it promotes the development of other RFs [59]. In some meta-analyses related to telemedicine, a reduction in factors associated with obesity, such as total cholesterol, LDL cholesterol, blood pressure control and glycemic control, has been observed [60,61,62]. However, the effects observed in the studies analyzed in this review and meta-analysis were smaller compared to those previously cited. This difference is attributed to the participants’ loss of interest in telemedicine-based treatment. Even so, it is clear that there are benefits for the health of the subject, in addition to generating a positive impact on the health system, such as cost reductions, monitoring of target patients, improvement and shortening of referrals to specialized medicine as well as accessibility for all patients and equity in care [53].

The overall effects of the telemedicine interventions showed good internal consistency: the reduction in obesity, hypercholesterolemia and glycosylated hemoglobin parameters coincided with better dietary and physical activity patterns [52]. Considering that one of the main aspects that influence the course of chronic diseases, including cardiovascular disease RFs, is the adherence to treatments [63], telemedicine may have its main effects in favoring the correct follow-up and the adoption of healthy lifestyle habits [64]. The constant reminder, the possibility of clarifying doubts and the direct contact with health professionals that overcome geographical barriers are some of the reasons why telemedicine is effective [21]. In addition, the possibility of a comprehensive intervention on RFs is relatively simple and inexpensive, being easily integrated into routine clinical practice, especially in countries with limited access to the Internet or few resources for health care [65].

Finally, it is expected that the applicability of telemedicine and its effects will potentially improve over time. This is mainly due to the familiarization of young populations with the technology and the incursion into the use of more advanced platforms with the aim of decentralizing medical care, as is the case of the metaverse [66].

The limitations of this review and meta-analysis are mainly related to the great heterogeneity existing among the intervention protocols based on telemedicine. The lack of a single intervention protocol with uniform criteria makes the replicability of the studies almost impossible; however, this variety, in turn, favors the individualization of the patient with the benefits that this entails. Additionally, another limitation of this review and meta-analysis is a possible publication bias, since only published articles were included while intervention protocols were not considered. This review can serve as a basis for future studies that explore in much greater depth each of the elements that make up cardiovascular risk.

## 5. Conclusions

Although the current evidence is heterogeneous regarding the statistically significant effects of telemedicine on several cardiovascular RFs, its clinical relevance is undeniable. Telemedicine interventions improve long-term risk factor control and body composition by improving adherence to treatments, allowing patients to have constant reminders and the possibility to clarify doubts and to be in contact with health professionals regardless of geographical barriers. It is necessary that telemedicine-based interventions and the means employed have been proven through clinical trials. Finally, the application of telemedicine-based interventions eliminates geographical barriers and increases patient accessibility to the health system with minimal risk, consolidating it as an important option in low-income countries.

## Figures and Tables

**Table 1:** Methodological quality of the articles included.

Article	1	2	3	4	5	6	7	8	9	10	11	Total
Frias et al., 2017 [20]	Y	Y	Y	Y	Y	Y	N	Y	Y	Y	Y	9
Benson et al., 2019 [21]	Y	Y	Y	Y	Y	Y	N	Y	Y	Y	N	8
Buysse et al., 2020 [22]	Y	Y	Y	Y	Y	N	N	Y	Y	Y	Y	8
Baron et al., 2017 [23]	Y	Y	Y	Y	N	N	N	Y	Y	Y	Y	7
Lee et al., 2020 [24]	Y	Y	Y	Y	Y	N	Y	Y	Y	Y	Y	9
Duruturk et al., 2019 [25]	Y	Y	N	Y	N	Y	Y	Y	Y	Y	Y	8
Jeong et al., 2018 [26]	Y	Y	N	Y	N	N	N	Y	Y	Y	Y	6
von Storch et al., 2019 [27]	Y	Y	N	N	N	N	Y	Y	Y	Y	Y	6
Oseran et al., 2022 [28]	Y	Y	Y	N	N	N	N	Y	Y	Y	Y	6
Warren et al., 2018 [29]	Y	Y	Y	N	N	N	Y	N	Y	Y	Y	6
Sarayani et al., 2018 [30]	Y	Y	Y	Y	N	N	Y	Y	Y	Y	Y	8
Franc et al., 2020 [31]	Y	Y	Y	N	N	N	Y	Y	Y	Y	Y	7
Parsons et al., 2019 [32]	Y	Y	Y	N	N	N	Y	Y	Y	Y	Y	7
Gong et al., 2020 [33]	Y	Y	N	Y	N	N	N	Y	Y	Y	Y	6
Nolan et al., 2018 [34]	Y	Y	Y	N	Y	N	Y	Y	Y	Y	Y	8
Kao et al., 2019 [35]	Y	Y	Y	Y	Y	N	N	Y	Y	Y	Y	8
Liu et al., 2020 [36]	Y	Y	N	N	Y	N	Y	Y	Y	Y	Y	7
Liu et al., 2018 [37]	Y	Y	Y	Y	Y	N	Y	Y	Y	Y	Y	9
Meurer et al., 2019 [38]	Y	Y	Y	N	N	N	N	Y	Y	Y	Y	6
Lugones-Sanchez et al., 2020 [39]	Y	Y	Y	N	N	N	Y	Y	Y	Y	Y	7
Thomas et al., 2017 [40]	Y	Y	N	N	N	N	Y	Y	Y	Y	Y	6
Alencar et al., 2019 [41]	Y	Y	N	N	N	N	N	N	N	Y	Y	3
Duncan et al., 2020 [42]	Y	Y	N	N	N	N	Y	Y	Y	Y	Y	6
Lugones-Sanchez et al., 2022 [43]	Y	Y	Y	Y	Y	N	N	Y	Y	Y	Y	8
Thomas et al., 2019 [44]	Y	Y	N	N	N	N	Y	Y	Y	Y	Y	6
Johnson et al., 2019 [45]	Y	Y	Y	N	Y	N	N	N	N	Y	Y	5
Rayward et al., 2020 [46]	Y	Y	Y	N	N	N	N	Y	Y	Y	Y	6
Murawski et al., 2019 [47]	N	Y	Y	Y	N	N	N	Y	Y	Y	Y	7

Items: 1 = eligibility criteria; 2 = random allocation; 3 = concealed allocation; 4 = baseline comparability; 5 = blind subjects; 6 = blind therapists; 7 = blind assessors; 8 = adequate follow-up (>85% of the subjects are assessed in each measurement point); 9 = intention-to-treat analysis; 10 = between-group comparisons; 11 = point estimates and variability. Y = Yes; N = No.

**Table 2:** Effects of telemedicine-based interventions in persons with diabetes.

Author and Year	Sample CG/IG	Control Group	Intervention Group				
			Intervention Type	HbA1c at Baseline (%)	Reduction in HbA1c over Time (%)		
Frias et al., 2017 [19]	29/80	Usual care	Telemonitoring	9.54 (SD = 0.19)	3 months −0.50 (SD = 0.20)	-	-
Benson et al., 2019 [20]	60/64	Usual care	Telemonitoring + e-consulting	8.1 (SD = 1.55)	3 months −0.21 (SD = 0.25)	-	-
Buysse et al., 2020 [21]	72/81	Usual care	Telemonitoring + e-consulting	8.30 (SD = 1.58)	-	-	24 months −0.40 (CI = −1–0.2) *
Baron et al., 2017 [22]	36/45	Usual care	Telemonitoring + e-consulting	9.07 (SD = 1.72)	3 months 8.76 (SD = 1.70)	9 months 8.56 (SD = 1.64)	-
Lee et al., 2020 [23]	120/120	Usual care	Telemonitoring	9.00 (CI = 8.97–9.03)	3 months −0.14 (CI = −0.24–0.04)	6 months −0.69 (CI = −0.74–−0.65)	12 months −0.33 (CI = −0.37–−0.29)
Duruturk et al., 2019 [24]	21/23	Usual care	Telemedicine	7.14 (SD = 0.91)	6 weeks −1.21 (SD = 0.78)	-	-
Jeong et al., 2018 [25]	113/113	Usual care	Telemedicine	8.39 (SD = 1.10)	-	6 months −0.66 (SD = 1.09)	-
von Storch et al., 2019 [26]	55/60	Usual care	Telemedicine + self-management program	7.05 (SD = 0.98)	3 months −0.46 (SD = 0.75) *	-	-
Oseran et al., 2022 [27]	130/130	Usual care	E-consulting with endocrinologist	9.50 (SD = 0.90)	6 months −0.83 (SD = 1.41)	12 months −0.75 (SD = 1.48)	18 months −0.85 (SD = 1.42)
Warren et al., 2018 [28]	63/63	Usual care	Telemedicine	8.40 (CI = 7.80–9.00)	-	6 months 7.5 (CI = 6.90–8.20)	-
Sarayani et al., 2018 [29]	44/40	Usual care	Telemonitoring	7.84 (SD = 1.17)	3 months 6.97 (SD = 1.14) *	9 months 6.96 (SD = 1.44)	
Franc et al., 2020 [30]	221/213	Usual care	Telemonitoring	9.10 (SD = 1.00)	-	-	12 months −0.51 (CI= −0.73–−0.30) *
Parsons et al., 2019 [31]	116/108	Usual care	Self-monitoring + telecare	8.60 (SD = 1.15)	3 months −0.47 (CI = −0.68–0.25) *	6 months −0.71 (CI = −0.98–−0.44) *	12 months −0.87 (CI = −1.19–0.55) *

CG = control group; IG = intervention group; SD = standard deviation; CI = 95% confidence interval. * Statistically significant.

**Table 3:** Effects of telemedicine-based interventions in persons with.

Author and Year	Sample CG/IG	Control Group	Intervention Group			
			Intervention Type	BP (sBP/dBP) at Baseline (mmHg)	Reduction in BP (sBP/dBP) over Time (mmHg)	
Gong et al., 2020 [33]	218/225	Usual care	Telemonitoring	141.10 (SD = 10.10)/ 82.50 (SD = 9.60)	-	6 months −8.90 (SD = 6.40) */ −7.00 (SD = 6.10) *
Nolan et al., 2018 [34]	131/133	Usual care	E-counseling	141.50 (CI = 139–143)/ 87.30 (CI = 86–89)	4 months −8.40 (CI = −10.80–−5.90)/ −3.90 (CI = −5.50–−2.40)	12 months −10.10 (CI = −12.50–−7.60)/ −4.90 (CI = −6.40–3.50)
Kao et al., 2019 [35]	111/111	Usual care	Website	142.90 (SD = 14.10)/ 84.40 (SD = 10.80)	3 months −5.28 (SD = 1.35) * / −1.58 (SD = 0.83)	6 months −7.99 (SD = 1.89) * / 0.47 (SD = 1.03)
Liu et al., 2020 [36]	131/133	Usual care	E-counseling	141.50 (CI = 139–143)/ 87.30 (CI = 86–89)	-	12 months −10.10 (CI = −12.50–−7.60)*/ Not reported
Liu et al., 2018 [37]	43/43	Usual care	E-counseling	140.30 (SD = 2.10)/ 89.4 (SD = 1.60)	4 months −11.90 (CI = −14.90–−9.10)/ −6.90 (CI = −8.80–−5.00)	-
Meurer et al., 2019 [38]	27/28	Usual care	E-counseling + Telecare	Not reported	4 months −8.70 (SD = 2.80) */ Not reported	-

CG = control group; IG = intervention group; sBP = systolic blood pressure; dBP = diastolic blood pressure; SD = standard deviation; CI = 95% confidence interval. * Statistically significant.

**Table 4:** Effects of telemedicine-based interventions in persons with overweight.

Author and Year	Sample CG/IG	Control Group	Intervention Group				
			Intervention Type	BW at Baseline (kg)	BW Loss over Time (kg)		
Lugones-Sánchez et al., 2020 [39]	29/80	Usual care	Telemedicine + telemonitoring + telecare	89.70 (SD = 13.10)	3 months −2.04 * (CI = −2.57–−1.50)	-	-
Thomas et al., 2017 [40]	60/64	Usual care	Telemonitoring	93.40 (SD = 14.00)	3 months −2.70 * (CI = −3.50–−2.00)	-	12 months −2.1 * (CI = −3.30–−1.10)
Alencar et al., 2019 [41]	72/81	Usual care	Telemonitoring	106.70 (SD = 25.50)	3 months −7.30 (SD = 4.40) *	-	-
Duncan et al., 2020 [42]	36/45	Usual care	Telemonitoring	90.80 (SD = 13.10)	-	6 months −3.51 (SD = 4.77)	12 months −3.59 (SD = 5.60)
Lugones-Sánchez et al., 2022 [43]	120/120	Usual care	Telemedicine	91.40 (SD = 14.80)	3 months −1.79 * (CI = −2.20–−1.37)	-	12 months −1.46 * (CI = −2.15–−0.77)
Thomas et al., 2019 [44]	21/23	Usual care	Telemedicine + telemonitoring +	95.90 (SD = 17.00)	6 months −7.2 (CI = −8.5–−5.9)	12 months −6.6 (CI = −8.0–−5.1)	18 months −5.5 (CI = −7.1–−3.9)
Johnson et al., 2019 [45]	113/113	Usual care	Telemonitoring + telecare	112.80 (SD = 25.80)	3 months −8.23 (SD = 4.50) *	-	-

CG = control group; IG = intervention group; BW = body weight; SD = standard deviation; CI = 95% confidence interval. * Statistically significant.

**Table 5:** Effects of telemedicine-based interventions in sedentary people.

Author and Year	Sample CG/IG	Control Group	Intervention Group			
			Intervention Type	MVPA at Baseline (min)	Difference in MVPA over Time (min)	
Rayward et al., 2020 [46]	29/80	Usual care	Telemonitoring	120 (CI = 50–120)	3 months 5.20 (CI = 5.01–5.39)	6 months 5.16 (CI = 4.94–5.37)
Murawski et al., 2019 [47]	60/64	Usual care	Telemonitoring	164.00 (SD = 165.45)	3 months 428.40 (SD = 523.41)	6 months 405.30 (SD = 491.45)

CG = control group; IG = intervention group; MVPA = moderate-to-vigorous-intensity physical activity; SD = standard deviation; CI = 95% confidence interval.

## Data Availability

Not applicable.
